# Antibacterial and Antifungal Activity of the Extracts of Different Parts of *Avicennia marina* (Forssk.) Vierh

**DOI:** 10.3390/plants10020252

**Published:** 2021-01-28

**Authors:** Mohammad K. Okla, Abdulrahman A. Alatar, Saud S. Al-amri, Walid H. Soufan, Altaf Ahmad, Mostafa A. Abdel-Maksoud

**Affiliations:** 1 Department of Botany, College of Science, King Saud University, Riyadh 11451, Saudi Arabia; aalatar@ksu.edu.sa (A.A.A.); saualamri@ksu.edu.sa (S.S.A.-a.); 2 Department of Plant Production, Faculty of Food and Agriculture Sciences, King Saud University, Riyadh 11451, Saudi Arabia; waoufan@ksu.edu.sa; 3 Department of Botany, Aligarh Muslim University, Aligarh 202002, India; 4 Department of Zoology, Faculty of Science, King Saud University, Riyadh 11451, Saudi Arabia

**Keywords:** *Avicennia marina*, antibacterial, antifungal, *Pseudomonas aeruginosa*, *Bacillus subtilis*, *Staphylococcus aureus*, *Escherichia coli*

## Abstract

Increased problems associated with side effects and bacterial resistance of chemical drugs has prompted the research focus on herbal medicines in the past few decades. In the present investigation, the antimicrobial activity of the various parts of *Avicennia marina* (AM), a mangrove plant, has been evaluated. The plants were collected from the Jazan area of the Kingdom of Saudi Arabia. Primary extracts of roots, stem, leaves, fruits, and seeds were made in ethanol and fractioned in ethanol, ethyl acetate, petroleum ether, chloroform, and water. Minimal inhibitory concentration (MIC) and minimal bactericidal concentration (MBC) of the extracts were determined against *Bacillus* *subtilis*, *Escherichia coli*, *Pseudomonas aeruginosa*, and *Staphylococcus aureus*. It has been observed that the chloroform extract of roots of the AM exhibited inhibitory effects against both *S. aureus* (MIC = 1.5 ± 0.03 mg/mL) and *E. coli* (MIC = 1.7 ± 0.01 mg/mL). The ethanolic extract of the AM roots has shown antibacterial activity against *Pseudomonas aeruginosa* (MIC = 10.8 ± 0.78 mg/mL)*, Bacillus* *subtilis* (MIC = 6.1 ± 0.27 mg/mL), *Staphylococcus aureus* (MIC = 2.3 ± 0.08 mg/mL), and *Escherichia coli* (MIC = 6.3 ± 0.28 mg/mL). The leaf extract of the AM in ethyl acetate showed antibacterial activity against *S. aureus* and *E. coli*. Antifungal activity of these extracts was also investigated against *Aspergillus fumigatus* and *Candida albicans.* Ethanolic extract of roots and seeds of the AM has shown antifungal activity against *Aspergillus fumigatus* when applied individually. Ethanolic extract of the AM fruits has shown an inhibitory effect on the growth of *Aspergillus fumigatus* and *Candida albicans*. It is suggested that the plant extracts of AM have tremendous antimicrobial activity against a group of microbes, and this effect depends on both the plant part and the solvent used for extraction. Therefore, this plant can be considered to treat various diseases caused by antibiotic-resistant bacteria.

## 1. Introduction

A critical issue in the present healthcare scenario is the exponential increase in the multidrug-resistant bacteria (MDR) against antibiotics. This increase in the MDR is one reason for the failure of the treatments and the higher mortality percentage [1]. Therefore, it is the need of the hour to develop such antibacterial agents that can check drug resistance and treat the infectious disease efficiently. Fungal resistance to antibiotics is also rising, demanding the development of new antifungal agents [2]. For example, *Candida albicans* and *Aspergillus fumigatus* are opportunistic fungi. These fungi can produce invasive fungal infections in any organ of humans [3]. Limited numbers of therapeutic antifungal agents are available for these fungi because of increased resistance mechanisms through the biofilms’ formation by the fungal strains [2].

It is well known that plant parts as a whole or their extracts in different solvents are applied for various health ailments since time immemorial. Since natural herbal remedies of diseases are the outcome of hundreds of years of careful evaluation of their therapeutic efficacy and risks, side effects, and properties of these herbal based treatments are well known. Further, the present consumers’ interest is for such natural foods that should be effective antimicrobial agents with no side effects [4]. Mangroves are a group of trees and shrubs that live in the coastal intertidal zone. Mangrove trees can grow under adverse environmental conditions like low-oxygen soil, high temperature, high salinity, etc. The distribution of mangrove forests is mainly at the tropical and subtropical latitudes [5]. Recently, many studies have directed towards investigating the biological activity of mangroves for the treatment of various diseases. Recently, Ser et al. [6] isolated two strains of *Streptomyces* from Peninsular Malaysia mangrove sediments. Bioactivity screening of these strains showed potent antioxidant and cytotoxic activities against human cancer cell lines [6]. Several bioactive compounds have been isolated and characterized from mangrove plants. These belong to the categories of glycosides, tannins, terpenes, steroids, naphthoquinones, alkaloids, and flavonoids [7]. Due to the presence of the varied levels of bioactive compounds, research interest in mangrove plants for their therapeutic activities, including antimicrobial effects, is increasing continuously [8].

*Avicennia marina* (Forssk.) Vierh., a mangrove tree belonging to the Acanthaceae family, is mostly found in the subtropical and tropical regions of the Indo-West-Pacific area. It is considered a representative example of mangroves that have been widely investigated for their medicinal importance [9]. This tree withstands severe environmental stresses [10,11]. It has been reported that *A. marina* can tolerate a very high level of salinity and temperature [12]. The survival capability of *A. marina* under severe environmental conditions is because of the presence of a large number of phytochemicals [13]. The presence of the various categories of phytochemicals makes this plant an excellent candidate for the treatment of various health ailments. *Avicennia marina* is used in the traditional medicine system for many centuries. For example, leaves are used for the treatment of ulcers, abscesses, and rheumatism. The leaf decoction is also used for the treatment of malarial fever and food poisoning [14]. Ringworms, skin ulcers, and scabies are treated using barks and fruits [15]. ElDohaji et al. [16] systematically reviewed the medicinal properties of *A. marina* and reported multiple health benefits. Various researchers are conducting studies on evaluating the medicinal properties of *A. marina* through in vitro experimentations. Evaluation of the antiviral activity of methanolic, ethanolic, aqueous, and n-hexane extracts of *A. marina* against HIV-1 and HSV have shown that the maximum antiviral activity was with the application of methanol extract [17]. Behbahani et al. [18] isolated luteolin-7-O-methyl ether-3’-O-β-d-glucoside from *A. marina,* and they showed antiherpetic activity of AM to this compound. It was speculated that this compound probably hinders the attachment of HSV to the cell membrane, and, thereby, HSV entry into the cell. Additional studies on the evaluation of the biological activity of the methanolic extract of *A. marina* seeds and its column chromatography-based fractions have reported an iridoid glycoside that restricts the replication of HIV-1 in the initial stage of infection [19]. Besides the antiviral activity, the antibacterial activity of AM extracts was also investigated [20,21]. However, a detailed investigation of antibacterial and antifungal activity of the extracts of different parts of *A. marina* in different solvent systems has not been evaluated. Given this, the present study has shown in vitro antibacterial and antifungal activity of fractions of ethanolic extracts of different parts of *A. marina* in various solvents (water, ethanol, ethyl acetate, petroleum ether, and chloroform) against *Pseudomonas aeruginosa, Bacillus subtilis*, *Staphylococcus aureus*, and *Escherichia coli* bacterial strains and *Aspergillus fumigatus, Candida albicans,* and *Mucor* sp. fungal strains.

## 2. Results

Preliminary screening of the extracts of various parts of AM in different solvents for antimicrobial activity revealed that only root and leaves exhibited antibacterial activity. Antifungal activity was shown by the root, fruit, and seeds. These parts of the AM were taken for further study.

### 2.1. Antibacterial Activity of Extract of the Roots of Avicennia marina

**Figure 1:**
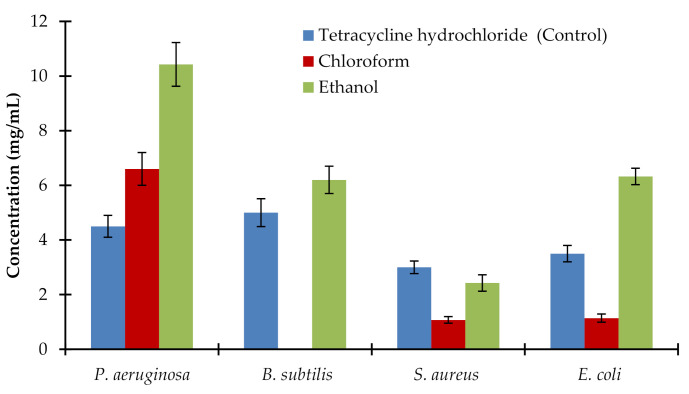
Minimal inhibitory concentration (MIC) values for ethanol and chloroform *Avicennia marina* roots extract against *P. aeruginosa, B. subtilis, S. aureus,* and *E. coli*. Tetracycline hydrochloride was a positive control. Aqueous, petroleum ether, and ethyl acetate extracts did not affect any of the tested bacterial strains. Chloroform extract did not affect *B. subtilis*. Values are the mean of five replicates (*n* = 5). Vertical bars show standard error.

Minimal inhibitory concentration (MIC) values were used to determine the susceptibilities of bacteria to drugs and also to evaluate the activity of new antimicrobial agents. As illustrated in Figure 1, using an agar diffusion method with commercially available strips containing an exponential gradient of antibiotic, the chloroform extract of AM roots exhibited the lowest MIC for *S. aureus* and *E. coli* with concentrations of 1.5 ± 0.03 mg/mL and 1.7 ± 0.01 mg/mL, respectively that was lower than the control values (3.0 ± 0.2 mg/mL and 3.5 ± 0.34 mg/mL, respectively). Antibacterial activity was also observed by the application of the ethanolic extract of AM roots. The MIC values for *P. aeruginosa, B. subtilis*, *S. aureus*, and *E. coli* were 10.8 ± 0.78, 6.1 ± 0.27, 2.3 ± 0.08, and 6.3 ± 0.28 mg/mL, respectively with the application of ethanolic extract when compared with the control values of 4.6 ± 0.22, 5.1 ± 0.25, 3.0 ± 0.2, and 3.5 ± 0.34, respectively. No significant effects of ethyl acetate, petroleum ether, and aqueous extracts of AM roots were reported on any of the tested bacterial strains.

### 2.2. Antibacterial Activity of the Leaf Extract of Avicennia marina

**Figure 2:**
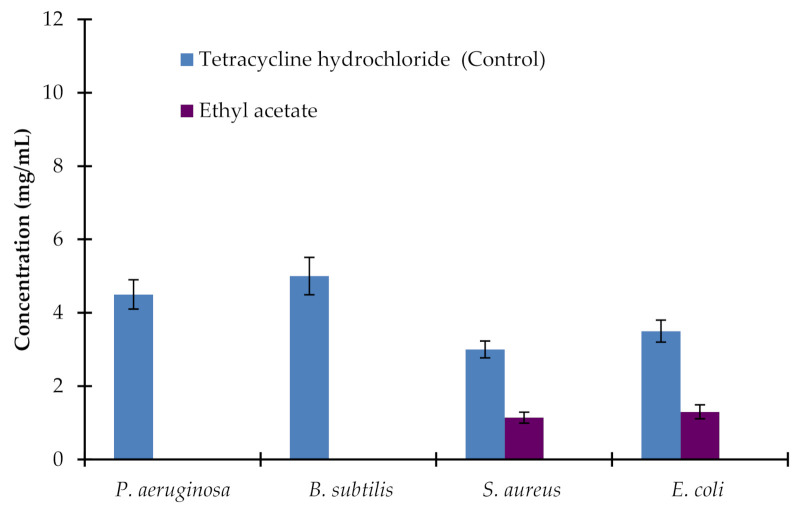
MIC values for ethyl acetate *Avicennia marina* leaf extract against *P. aeruginosa, B. subtilis, S. aureus,* and *E. coli*. Tetracycline hydrochloride was a positive control. Aqueous, chloroform, ethanol, and petroleum ether extracts did not affect any of the tested bacterial strains. Ethyl acetate extract did not affect *P. aeruginosa* and *B. subtilis*. Values are the mean of five replicates (*n* = 5). Vertical bars show standard error.

Among the different solvent extracts of the leaves of AM, significant antibacterial activity was observed by applying the extract of leaves with ethyl acetate. The MIC values for the ethyl acetate extract of AM leaves against *S. aureus*, and *E. coli* were 1.2 ± 0.1 mg/mL and 1.4 ± 0.15 mg/mL, compared to the MIC values of 3.0 ± 0.21 and 3.5 ± 0.29 mg/mL, respectively of the control. On the other hand, all ethanol, petroleum ether, chloroform, and aqueous extracts had no significant effects on any tested bacterial strains (Figure 2).

### 2.3. Minimal Bactericidal Concentration for Extract of the Roots of Avicennia marina

**Figure 3:**
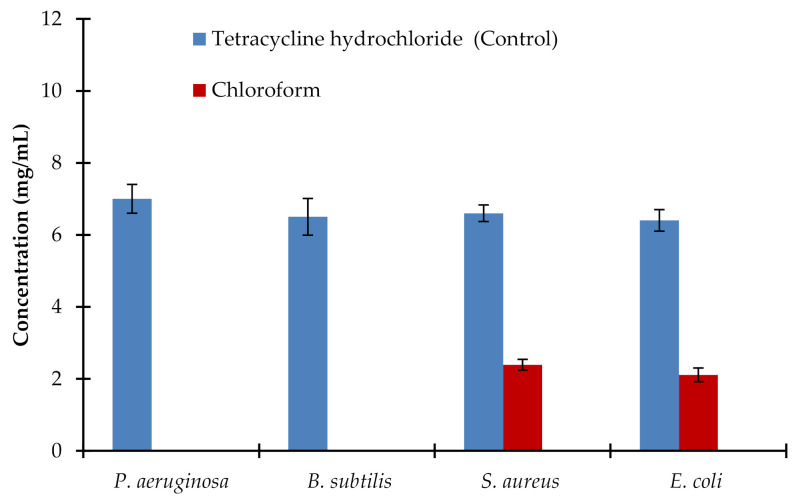
MBC values for chloroform *Avicennia marina* roots extract against *P. aeruginosa, B. subtilis, S. aureus,* and *E. coli*. Tetracycline hydrochloride was a positive control. Aqueous, ethanol, petroleum ether, and ethyl acetate extracts did not affect any tested bacterial strains. Chloroform extract did not affect *P. aeruginosa* and *B. subtilis*. Values are the mean of five replicates (*n* = 5). Vertical bars show standard error.

The minimum bactericidal concentration (MBC) could be used as a confirmatory parameter for the antibacterial activity of any drug. As illustrated in Figure 3, the MBC was reported by applying the chloroform extract of the root of the AM. The values of MBC for the chloroform extract of AM roots against *S. aureus* and *E. coli* were 3.2 ± 0.09 mg/mL and 2.9 ± 0.11 mg/mL, respectively in comparison to the control (6.6 ± 0.31 and 6.4 ± 0.39) mg/mL. No MBC was observed by applying extracts of the roots of AM in ethanol, petroleum ether, ethyl acetate, and water on the tested bacterial strains.

### 2.4. Minimal Bactericidal Concentration for Extracts of Leaves of Avicennia marina

**Figure 4:**
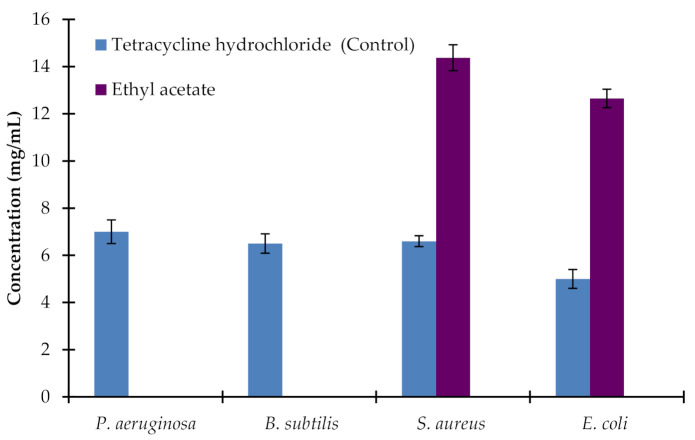
MBC values for ethyl acetate *Avicennia marina* leaf extract against *P. aeruginosa, B. subtilis, S. aureus,* and *E. coli*. Tetracycline hydrochloride was a positive control. Aqueous, ethanol, petroleum ether, and chloroform extracts did not affect any tested bacterial strains. Ethyl acetate extract did not affect *P. aeruginosa* and *B. subtilis*. Values are the mean of five replicates (*n* = 5). Vertical bars show standard error.

As illustrated in Figure 4, significant minimal bactericidal concentration (MBC) was reported with the application of ethyl acetate extract of the leaves of AM. The MBC for the ethyl acetate extract of AM leaves against *S. aureus* and *E. coli* were 14.2 ± 0.5 mg/mL and 12.5 ± 0.32 mg/mL, as compared to the control MBC values of 6.3 ± 0.21 and 5.1 ± 0.19 mg/mL, respectively. Extract of leaves in ethanol, petroleum ether, chloroform, and water did not significantly affect any of the tested bacterial strains.

### 2.5. Antifungal Activity of Extract of Different Parts of Avicennia marina

**Figure 5:**
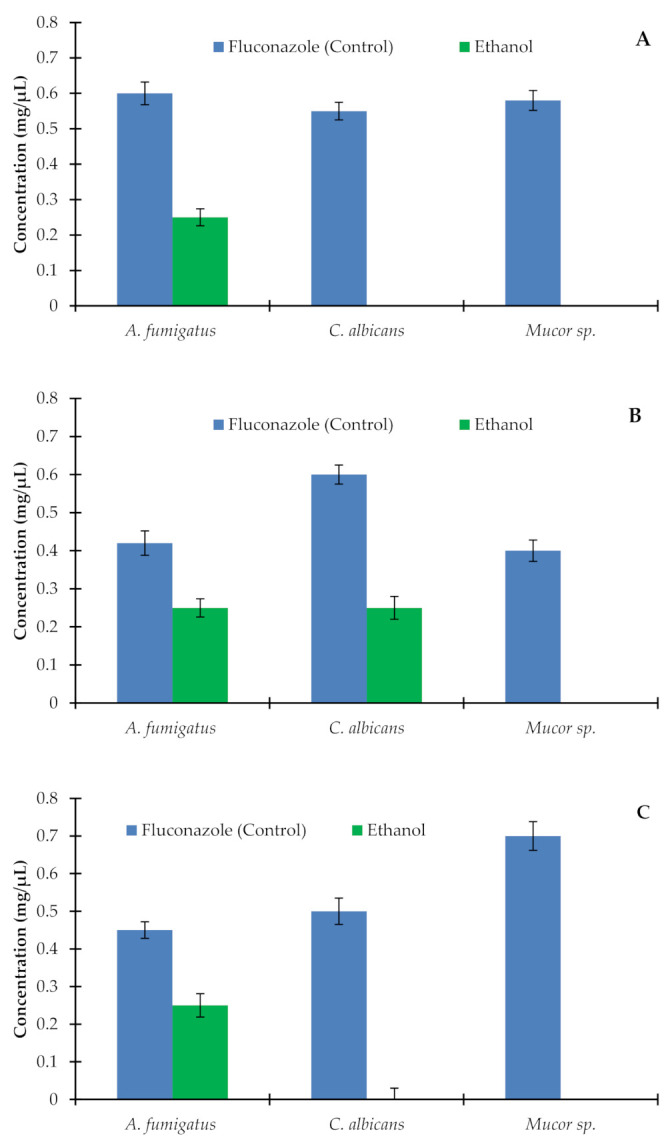
MIC values for the ethanolic extract of the root (**A**), fruits (**B**), and seeds (**C**) of *Avicennia marina* against *Aspergillus fumigatus*, *Candida albicans,* and *Mucor* sp. Fluconazole was used as the control. Ethyl acetate, petroleum ether, chloroform, and aqueous extracts of root, fruit, and seeds of *Avicennia marina* did not show any inhibitory effects on the tested fungal strains. Only ethanol extract showed an inhibitory effect on *A. fumigatus* and *C. albicans.* Values are the mean of five replicates (*n* = 5). Vertical bars show standard error.

Antifungal activity was also examined to evaluate the biological activity of AM on different types of fungal strains, viz. *Aspergillus fumigatus*, *Candida albicans*, and *Mucor* sp. Extracts of roots, fruits, and seeds were carried out using ethanol, ethyl acetate, petroleum ether, chloroform, and water, and antifungal activity of these extracts was monitored on three different fungal strains (Figure 5). Among the extracts of the AM root in different solvents, antifungal activity was observed by the application of ethanolic extract on *Aspergillus fumigatus* only. Ethanolic extract of the AM root did not show the antifungal effect on *Candida albicans* and *Mucor* sp. The MIC value was lower (0.25 ± 0.01 mg/mL) than that of the control fluconazole (0.6 ± 0.03 mg/mL). Extracts of the AM root in ethyl acetate, petroleum ether, chloroform, and water did not show any antifungal effects on any fungal strain (Figure 5a). Among the extracts of AM fruits in different solvents, the antifungal effect was reported by applying the ethanolic extract that showed an antifungal effect against *Aspergillus fumigatus* and *Candida albicans***.** The MIC values of 0.26 ± 0.02 mg/mL and 0.25 ± 0.01 mg/mL of *Aspergillus fumigatus* and *Candida albicans,* respectively, were lower than the control values of 0.42 ± 0.2 mg/mL and 0.6 ± 0.31 mg/mL, respectively (Figure 5b). Among the extracts of the seeds of AM in different solvents, only ethanolic extract showed antifungal activity. This antifungal activity was limited to the *A. fumigatus* with a MIC value of 0.26 ± 0.01 mg/mL lower than the control (0.45 ± 0.02 mg/mL) (Figure 5c).

### 2.6. Electron Microscopic Studies

**Figure 6:**
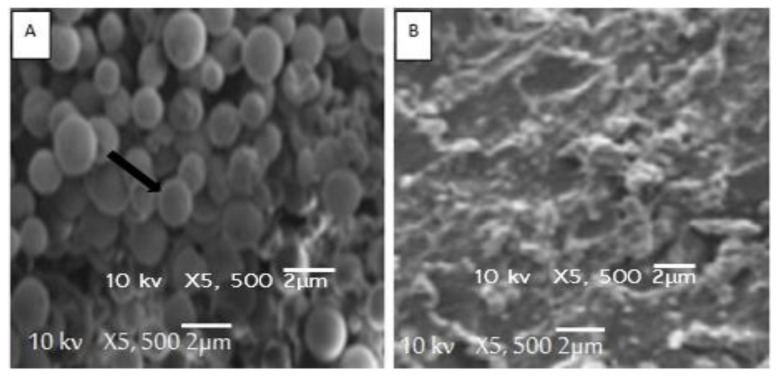
Scan electron microphotographs of untreated (**A**) and treated (**B**) *Candida albicans.*

**Figure 7:**
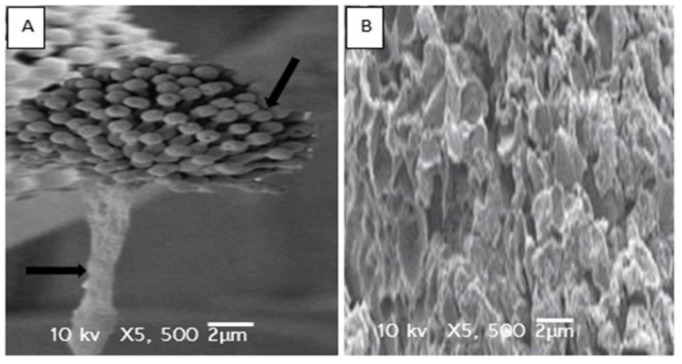
Scan electron microphotographs of untreated (**A**) and treated (**B**) *Aspergillus fumigatus*.

Antifungal activity for the ethanolic extract of AM fruits was further validated through image analysis of the treated and untreated fungal colonies using the scanning electron microscope (SEM). Micrographs of the colony of *Candida albicans*, treated with the ethanolic extract of the fruits of AM, showed a ruptured, incorporated, and shrinkage fungal hypha (Figure 6b). Contrary to this, the fungal hypha of the untreated colonies of the *Candida albicans* was normal in structure and shape (Figure 6a). Similarly, the colonies of the *Aspergillus fumigatus* (Figure 7b), treated with the ethanolic extract of the fruits of the AM, showed cytolysis and loss of cell integrity, compared to that of the untreated colony (Figure 7a).

## 3. Discussion

Humans have bee using plants for the treatment of various infectious diseases since ancient times. Scientific research for proving the therapeutic efficacy of a large number of medicinal plants are undergoing. Today, medicinal plants are being used in many countries for the treatment of different infectious diseases. The current interest in medicinal plants as therapeutic agents has emerged in various parts of the world because of the increasing incidence of drug-resistant bacteria and the appearance of new pathogenic bacteria strains. In vitro testing of a large number of plants has been carried out against different bacterial strains, and it has been reported that extracts and pure compounds of many medicinal plants are very effective against bacterial strains [22]. Many scientific reports have shown potential foliar extracts of mangrove against microbial pathogens and suggested considering the mangrove plants as a valuable source for the bioactive chemicals of immense medicinal values [23,24,25]. The present study has shown in vitro experimental evaluation of the effectiveness of antibacterial activity of extracts of plant parts of *A. marina* (a mangrove tree) in different solvent systems against *Bacillus subtilis*, *Escherichia coli*, *Pseudomonas aeruginosa*, and *Staphylococcus aureus*. The antifungal activity of these extracts was also evaluated against *Aspergillus fumigatus* and *Candida albicans.* This study reported that the ethanolic extracts of AM roots showed antibacterial effects against all the four tested bacteria, viz., *B. subtilis*, *E. coli*, *P. aeruginosa,* and *S. aureus*. Root extract in chloroform showed antibacterial activity against *E. coli*, *P. aeruginosa,* and *S. aureus*. The root extracts in other solvents did not show antibacterial activity on the tested bacteria. These observations are in agreement with earlier reports showing the antibacterial activity of the alcoholic and the chloroform root extracts of AM [26]. While the extract of leaves of AM in ethyl acetate exhibited antibacterial activity against *S. aureus* and *E. coli*, none of the extracts in ethanol, petroleum ether, chloroform, and water showed antibacterial activity in our study. Active components present in the plant extracts are responsible for the differential antibacterial activity as *Avicennia marina* accumulated many phytochemicals for its survival under severe environmental conditions. The phytochemicals belong to phenolics, steroids, triterpenes, glycosides, esters, aliphatic alcohols, amino acids, carotenoids, etc. [27,28]. The GC–MS based analysis of the chemical composition of extracts of plants parts in different solvent systems revealed the presence of some notable compounds like 1,2-Benzenedicarboxylic acid, Cis-cinnamic acid, hexadecanoic acid, 2,6,10,14,18,22-tetracosahexae, 25-ethyl-27-norcholesta-5,24(Z), 1-tetradecene, taraxasterol, hydroxymethylfurfural (HMF), and 1-deoxy-D-altritol (Supplementary Table S1). These compounds have proven antimicrobial activity. Khattab and Temraz [29] also reported 2-propenoic acid, 3-phenyl ester, 3-acetyl methoxyphenyl, benzaldehyde, 3-hydroxyl-4-methoxy, and 1,2-benzenediol in the leaf extract of *A. marina* and phosphonic acid and p-hydroxyphenyl in the flower extract. Naphthofuran compounds like naphtho[1,2-b]furan-4,5-dione, 2-[2′-2′-hydroxypropyl]-naphtho[1,2-b] furan-4,5-dione, and 3-hydroxynaphtho[1,2-b] furan-4,5-dione were identified by Sutton et al. [30]. Isoverbascoside and D-rhamnosyl verbascoside were isolated from the leaves of *A. marina* [31]. Some important sterols like stigmasterol-3-O-β-D galactopyranoside [32], stigmasterol [33], β-sitosterol, and ergost-6,22-diene-5,8-epidioxy-3 β-ol [34] were also identified from the aerial roots and leaves of this plant. Wu et al. [35] extensively reviewed the worldwide mangrove species and summarized 349 metabolites of these plants in terms of source, chemistry, and bioactivity. The presence of identified chemical compounds in the extracts of plant parts of *A. marina* in different solvent systems in our study also matched with the compounds reported earlier in other mangrove plants. The chemical properties of the solvent used in the extraction process played a crucial role in exhibiting the plants’ antibacterial properties. The presence of these phytochemicals in the solvents may be correlated with the differential antibacterial activity against the bacterial strains. Ethanolic extracts of the roots proved the most effective in almost all the antibacterial tests, followed by the chloroform, indicating that maximum antibacterial active ingredients of *A. marina* roots were extracted into ethanol. It is suggested that the inability of the extracts of plant parts in other solvent systems to exhibit antibacterial activity against tested bacterial strains maybe because these strains of bacteria might have a kind of resistance mechanisms, e.g., modification of target sites, inactivation of enzymes, and reduced accumulation of drug, or the quantity of the bioactive compounds is very low [36]. Since control showed no inhibition, it proves that solvents did not act as antibacterial agents. Leaf extract of *A. marina* in ethyl acetate only showed antibacterial activity in our study. The presence of phytol (a constituent of chlorophyll, which could be converted to phytanic acid) in the leaves extract could be responsible for the antibacterial effects. Similarly, Raut and Anthappan [37] have documented antibacterial activity for the methanolic leaves extract of the mangrove, *Sonneratia alba,* and the effect was obvious for both *S. aureus* and *E. coli* among the different tested bacterial strains.

Analysis of the antifungal activity of the *A. marina* in our study indicated that the extract of the roots, fruits, and seeds of *A. marina* showed antifungal activity against *Aspergillus fumigatus* and *Candida albicans.* Khafagi et al. [38] has also reported antifungal activity of both aqueous and ethanol extracts of roots, cotyledons, leaves, and stems of *Avicennia marina.* Taken together, our data indicated that mangroves in general and *Avicennia marina,* in particular, have potent biological activity against an extensive array of microorganisms. These effects are primarily dependent on the plant parts and the solvent used or the extraction process.

The most common mode of the action of different bioactive compounds that are used to treat microbial infections is the interaction with the microbial enzyme system, interference with nucleic acids, interference with the cell membrane and cell wall, etc. [39,40,41,42]. Benzoic acid and phthalate, as identified in this study in the ethanolic extract of *A. marina*, have been reported to have a permeability barrier provided by the cells membrane, which is indispensable to many cellular functions, including maintaining the energy status of the cell, membrane coupled energy transducing process, solute transport, and metabolic regulation [43,44]. Stigmasterol, another compound of the plant extract of *A. marina*, has been reported to act as a β-lactamase inhibitor, which restores susceptibility of the antibiotic resistant bacteria to antibiotics [45]. Cinnamic acid and its hydroxylated derivatives have also been reported to have antifungal activity through their ability to prevent fungal spore germination and antityrosinase enzyme activity [46]. It has also been reported that the cinnamic acids caused fungal growth inhibition by interacting with benzoate 4-hydroxylase, an enzyme responsible for aromatic detoxification [47]. Hydroxymethylfurfural (HMF) has also been reported in the extracts of *A. marina*. The HMF is a honey component where it works as an antimicrobial agent, possibly because of its high osmolarity and acidity (low pH) [48,49]. Johannes et al. [50] reported that hexadecanoic acid reacted with the hydroxyl group of lipopolysaccharide, a component of the bacterial cell wall, resulting in the conversion of lipopolysaccharide membrane structure into the asymmetric form. As a result, the balance in the membrane lipid structure is disturbed, causing perturbance in the cell membrane. This change in the cell membrane resulted in the cell swelling and cytoplasm membrane damage, distended, and lysed. OH group of hexadecanoic acid has been reported to be toxic to the cell protoplasm. It infiltrates and damages the cell wall. It also causes denaturation of the cytoplasm protein Radiati [51].

## 4. Materials and Methods

### 4.1. Plant Material and Preparation of Extracts

Root, stem, leaves, stems, fruits, and seeds of *Avicennia marina* (Forssk.) Vierh. (*Avicenniaceae*) were collected from the mangrove stand of Jazan area that is located in the southern region of Saudi Arabia (Farasan Island, 16°42′21″ N 41°59′0″ E). The collected material was taxonomically identified, and the voucher specimen was kept in the herbarium of the Botany and Microbiology Department, College of Science, King Saud University, Saudi Arabia (Voucher No.: 23638). These plant parts were separately washed thoroughly with seawater to remove epiphytes, shells, and various extraneous matters. The cleaned plant parts were packed separately in polyethylene bags and brought to the laboratory. The collected samples were air-dried under shade. The dried plant parts were powdered separately, and homogenous powder of each plant part was obtained. Each plant part (100 g) was extracted with 95% 1000 mL ethanol with stirring for 72 h. Filtration of the extract was then carried out using a muslin cloth, centrifuged at 9000 rpm for 10 min. To obtain clear filtrate, the extract was filtered again using Whatman filter paper No. 41. The filtrates were evaporated and dried using a vacuum rotary evaporator to obtain the active ingredients in their solid phase. These solid active ingredients were then dissolved in ethanol solution (ethanol: water, 4:6, *v*/*v*). The sequential separation was performed with petroleum ether, ethyl acetate, ethanol, chloroform, and water to yield the respective solvent fractions. These fractions were then concentrated to get dried extract for further analysis of biological activities.

### 4.2. Microbial Strains and Culture Media

Four different pathogenic bacterial strains: *Bacillus subtilis* (ATCC 10400), *Escherichia coli* (ATCC 442), *Pseudomonas aeruginosa* (ATCC 27853), and *Staphylococcus aureus* (ATCC 29213), were used for this study. *Staphylococcus aureus* and *B. subtilis* are Gram-positive bacteria. *Escherichia coli*, *S. sonnei,* and *P. aeruginosa* are Gram-negative bacteria. These bacterial strains were procured from the Botany and Microbiology Department of King Saud University, Saudi Arabia. Fungal strains were *Aspergillus fumigatus* (ATCC46645), *Candida albicans* (ATCC28121), and *Mucor* sp. (Clinical isolates). These fungal strains were procured from the military hospital in Riyadh, Saudi Arabia. The bacterial species were grown in Mueller–Hinton agar (Merck, USA). Fungal species were grown in the Sabouraud dextrose broth medium.

### 4.3. Preparation of Inoculums

Each strain of bacteria from the stock cultures was streaked on the agar plate. The plate was then incubated for 24 h at 37 °C. Three bacterial colonies from each plate were emulsified in sterile 0.9% NaCl (*w*/*v*) to obtain 10^8^ CFU per mL (0.5 McFarland scale) as inoculums. The working bacterial suspension for the disk diffusion test was prepared through dilution with 0.9% sterile NaCl (*w*/*v*) until 10^7^ CFU per mL was reached. Similarly, fungal suspensions were adjusted to 10^7^ cells/mL.

### 4.4. Analysis of Antibacterial Activity

Evaluation of the antimicrobial activity of plant extracts was carried out using the disk diffusion method. Sterile filter paper disks (6 mm in diameter) were prepared and placed in Petri dishes. Fifty micrograms of residues of plant extract were redissolved separately. After sterilization, through a 0.22 mm Millipore filter, the plant extracts were loaded over filter paper discs, and a final concentration of 10 mg/mL was maintained. Mueller–Hilton agar was poured into Petri dishes. Each Petri dish was loaded with 150 µL of microbial suspension (1 × 10^16^ cells/mL) and then with filter paper discs containing plant extracts of different plant parts. One filter paper disc containing 5 mg of tetracycline hydrochloride (European Pharmacopoeia Reference Standard, Sigma-Aldrich, St. Louis, MO, USA), and one filter paper disc containing only the solvent (without plant extract) were also placed on the top of Mueller–Hilton agar plates in separate Petri dishes for positive control and negative control, respectively. The solvent for tetracycline hydrochloride was dimethyl sulfoxide. All the Petri dishes were kept at 5 °C for 2 h for the diffusion of plant extract and then incubated at 37 °C for 24 h anaerobically. Incubation results were monitored.

### 4.5. Determination of Minimum Inhibitory Concentration (MIC)

Minimum inhibitory concentration (MIC) of plant extracts that exhibiting a strong antimicrobial activity was evaluated against different bacterial strains. Sterilized extracts of plants parts in various solvents at a final concentration of 0.25, 0.75, 1.5, 2.5, 5.0, 7.5, 10.0, 12.5, and 15.0 mg/mL were loaded over filter paper discs. Mueller–Hilton agar was poured into Petri dishes. Each Petri dish was loaded with 150 µL of microbial suspension (1 × 10^16^ cells/mL), and then with filter paper discs containing plant extracts of different plant parts in various solvents. One filter paper disc containing 5 mg of tetracycline hydrochloride and one filter paper discs containing only the solvent (without plant extract) were also placed on the top of Mueller–Hilton agar plates in separate Petri dishes for positive control and negative control, respectively. All the Petri dishes were kept at 5 °C for 2 h for the diffusion of plant extract and then incubated at 37 °C for 24 h anaerobically. The MIC was determined as the minimum concentration of the plant extract that inhibits bacterial growth.

### 4.6. Determination of Minimum Bactericidal Concentration (MBC)

For the five different bacterial strains used, the MBC of plant extract was determined following the method described previously [52]. Bacterial streaks of the Petri dishes in which plants showed an antibacterial effect were taken from the inhibition zone of MIC plates. These bacterial streaks were placed on TSA (tryptone soya agar) plates and incubated at 35 °C for 24 h. Bacterial growth was examined in each Petri dish. The MBC was recorded as the concentration of extract of plant parts in different solvents that showed no bacterial growth.

### 4.7. Analysis of Antifungal Activity and Minimum Fungicidal Concentration

*Aspergillus fumigatus* (ATCC46645), *Candida albicans* (ATCC28121), and *Mucor* sp. were grown in Sabouraud dextrose (SD) broth at 27 °C for 48 h. The growth of the fungal strains was adjusted to OD_600_ of 0.1. Extracts of plant parts were filtered through 0.45 μm Millipore filters. SD agar was placed on several Petri dishes. Of suspension 100 μL containing 1.0 × 10^8^ CFU/mL of each fungal strain was spread on several Petri dishes. Filter paper disks (7 mm) were impregnated with 0.1, 0.2, 0.3, 0.4, 0.5, 0.6, 0.7, 0.8, 0.9, and 1.0 mg/mL extract of plant parts. One set of the disc was kept as negative controls in which only the solvents were used without plant extracts. One set of the disc was used as a positive control in which fluconazole (≥98% pure, Sigma-Aldrich, St. Louis, MO, USA) was added in place of plant extracts. The solvent for fluconazole was dimethyl sulfoxide. The inoculated Petri dishes were incubated at 27 °C for 72 h. Antifungal activity was monitored by analysis of the inhibition zone.

Minimum fungicidal concentration (MFC) was determined by adding different concentrations of plant extracts (0.1–1 mg/mL) were incorporated in SD broth in tubes. Of fungal suspension 1 mL was added to each tube. Incubation was carried out at room temperature for three days. Negative control was made in one tube that contained only the solvents. One tune was made as a positive control in which fluconazole was added in place of plant extracts. The lowest concentration of the plant extract inhibiting the growth of the fungus is regarded as the MFC.

### 4.8. Scan Electron Microscopic Analysis

For electron microscopic analysis, samples were prepared and investigated under the scanning electron microscope (JSM-7610F, JEOL, Tokyo, Japan), located in the Research Center of College of Science, King Saud University, Saudi Arabia.

### 4.9. Statistical Analysis

Data were subjected to the statistical analysis using the one-way analysis of variance (ANOVA). Means were separated by the least significant difference test (LSD, *p* ≤ 0.05) using the SPSS 21.0 statistical program “SPSS Inc., IBM” (Chicago, IL, USA). All measurements were performed five times for each treatment, and the means were reported.

## 5. Conclusions

Increasing resistance of the bacteria and fungi towards various antimicrobial synthetic drugs poses a major concern in medical science. It is well established from the ethnopharmacological investigations that the plants possess biologically active antimicrobial compounds. The present study has shown that extracts of root and leaves of *A. marina* exhibited antibacterial activity. Ethanolic extract of root proved to be the most effective against bacterial strains, *P. aeruginosa, B. subtilis*, *S. aureus*, and *E. coli* and fungal strains *Aspergillus fumigatus* and *Candida albicans.* Leaf extract in ethyl acetate showed significant antibacterial activity against *S. aureus* and *E. coli*. The antifungal activity has also been shown by the ethanolic extracts of fruit and seeds. The extracts of this plant were effective against *S. aureus*, known for its increased resistance to antibiotics. Therefore, this plant can be used to treat various diseases caused by antibiotic-resistant bacteria. There are a lot of potentials for this mangrove plant to treat infections caused by antibiotic-resistant bacteria. However, further research is required to understand the mechanisms involved in antimicrobial activity.

## Supplementary Materials

The following are available online at https://www.mdpi.com/2223-7747/10/2/252/s1. Table S1: GC-MS based analysis of chemical composition of various plant parts of *Avicennia marina* plant by the various solvents.

## References

[1] Nascimento G.G.F., Locatelli J., Freitas P.C., Silva G.L. (2000). Antibacterial activity of plant extracts and phytochemicals on antibiotic-resistant bacteria. Braz. J. Microbiol..

[2] Favre-Godal Q., Dorsaz S., Queiroz E.F., Marcourt L., Ebrahimi S.N., Allard P.M., Sanglard D. (2015). Anti-Candida Cassane-Type Diterpenoids from the Root Bark of *Swartzia simplex*. J. Nat. Prod..

[3] Soberón J.R., Lizarraga E.F., Sgariglia M.A., Juárez M.B.C., Sampietro D.A., Altabef A.B., Vattuone M.A. (2015). Antifungal activity of 4-hydroxy-3-(3-methyl-2-butenyl) acetophenone against *Candida albicans*: Evidence for the antifungal mode of action. Antonie van Leeuwenhoek.

[4] Siro I., Kapolna E., Kápolna B., Lugasi A. (2008). Functional food product development, marketing and consumer acceptance—A review. Appetite.

[5] Turner M. (2015). Ecology: Mangrove maintenance. Nature.

[6] Ser H.L., Tan L.T., Law J.W., Chan K.G., Duangjai A., Saokaew S., Pusparajah P., Ab-Mutalib N., Khan T.M., Goh B.H. (2017). Focused review: Cytotoxic and antioxidant potentials of mangrove-derived *Streptomyces*. Front. Microbiol..

[7] Haq M., Sani W., Hossain A., Taha R.M., Monneruzzaman K. (2011). Total phenolic contents, antioxidant and antimicrobial activities of *Bruguieragymnorrhiza*. J. Med. Plants Res..

[8] Abeysinghe P.D. (2010). Antibacterial activity of some medicinal mangroves against antibiotic resistant pathogenic bacteria. Indian J. Pharm. Sci..

[9] Bandaranayake W.M. (1998). Traditional and medicinal uses of mangroves. Mangroves Salt Marshes.

[10] Das S.K., Patra J.K., Thatoi H. (2016). Antioxidative response to abiotic and biotic stresses in mangrove plants: A review. Int. Rev. Hydrobiol..

[11] Thatoi H., Samantaray D., Das S.K. (2016). The genus *Avicennia*, a pioneer group of dominant mangrove plant species with potential medicinal values: A review. Front. Life Sci..

[12] Moore G.E., Grizzle R.E., Ward K.M., Alshihi R.M. (2015). Distribution, pore-water chemistry, and stand characteristics of the mangroves of the United Arab Emirates. J. Coast. Res..

[13] Osman N.A., Abkar F.A. (2016). Comparative evaluation of some selected bioactive constituents in the leaves and bark of *Avicennia marina* (Forsk.) Veirh. from the Sudanese red sea coast. J. Forest Prod. Indust..

[14] Rasoanaivo P., Petitjean A., Ratsimamanga-Urverg S., Rakoto-Ratsimamanga A. (1992). Medicinal plants used to treat malaria in Madagascar. J. Ethnopharmacol..

[15] Zhu F., Chen X., Yuan Y., Huang M., Sun H., Xiang W. (2009). The chemical investigations of the mangrove plant *Avicennia marina* and its endophytes. Open Nat. Prod. J..

[16] ElDohaji L.M., Hamoda A.M., Hamdy R., Soliman S.S.M. (2020). *Avicennia marina* a natural reservoir of phytopharmaceuticals: Curative power and platform of medicines. J. Ethnopharmacol..

[17] Namazi R., Zabihollahi R., Behbahani M., Rezaeic A. (2013). Inhibitory activity of *Avicennia marina*, a medicinal plant in Persian folk medicine against HIV and HSV. Iran. J. Pharm. Res..

[18] Behbahani M., Zadeh M.S., Mohabatkar H. (2013). Evaluation of antiherpetic activity of crude extract and fractions of *Avicenna marina*, in vitro. Antivir. Res..

[19] Behbahani M. (2014). Evaluation of anti-HIV-1 activity of a new iridoid glycoside isolated from *Avicenna marina*, in vitro. Int. Immunopharmacol..

[20] Devi A.S., Rajkumar J. (2013). In vitro antibacterial activity and stability of Avicennia marina against urinary tract infection pathogens at different parameters. Pak. J. Biol. Sci..

[21] Behbahani A., Shahidi F., Yazdi F.T., Mortazavi S.A., Mohebbi M. (2017). Use of *Plantago major* seed mucilage as a novel edible coating incorporated with *Anethum graveolens* essential oil on shelf life extension of beef in refrigerated storage. Int. J. Biol. Macromol..

[22] Mahady G., Huang Y., Doyle B., Locklear T. (2008). Natural products as antibacterial agents. Stud. Nat. Prod. Chem..

[23] Thatoi H.N., Patra J.K., Das S.K. (2014). Free radical scavenging and antioxidant potential of mangrove plants: A review. Acta Physiol. Plant..

[24] Gurudeeban S., Ramanathan T., Satyavani K. (2013). Antimicrobial and radical scavenging effects of alkaloid extracts from *Rhizophora zucronata*. Pharm. Chem. J..

[25] Mouafi F.E., Abdel-Aziz S.M., Bashir A.A., Fyiad A.A. (2014). Phytochemical analysis and antimicrobial activity of mangrove leaves (*Avicenna marina* and *Rhizophora stylosa*) against some pathogens. World Appl. Sci. J..

[26] Aruna K., Pendse A. (2012). Study on antibacterial activity of root extract from mangrove plants. Asian Sci..

[27] Li Y., Liu J., Yu S., Proksch P., Gu J., Lin W. (2010). TNF-α inhibitory diterpenoids from the Chinese mangrove plant *Excoecaria agallocha* L.. Phytochemistry.

[28] Okla M.K., Alamri S.A., Alatar A.A., Hegazy A.K., Al-Ghamdi A.A., Ajarem J.S., Faisal M., Abdel-Salam E.M., Ali H.M., Salem M.Z. (2019). Antioxidant, hypoglycemic, and neurobehavioral effects of a leaf extract of *Avicennia marina* on autoimmune diabetic mice. Evid. Based Complement. Alternat. Med..

[29] Khattab R.A., Temraz T.A. (2017). Mangrove *Avicennia marina* of Yanbu, Saudi Arabia: GC-MS constituents and mosquito repellent activities. Egypt. J. Aquat. Biol. Fish..

[30] Sutton D.C., Gillan F.T., Susic M. (1985). Naphthofuranone phytoalexins from the grey mangrove, *Avicennia marina*. Phytochemistry.

[31] Fauvel M., Taoubi K., Gleye J., Fouraste I. (1993). Phenylpropanoid glycosides from *Avicennia marina*. Planta Med..

[32] Mahera S., Saifullah S., Ahmad V., Mohammad F. (2013). Phytochemical studies on mangrove *Avicennia marina*. Pak. J. Bot..

[33] Mahera S., Ahmad V., Saifullah S., Mohammad F., Ambreen K. (2011). Steroids and triterpenoids from grey mangrove *Avicennia marina*. Pak. J. Bot..

[34] Jia R., Guo Y.-W., Hou H.X. (2004). Studies on the chemical constituents form leaves of *Avicennia marina*. Chin. J. Nat. Med..

[35] Wu J., Xiao Q., Xu J., Li M.-Y., Pana J.-Y., Yang M. (2008). Natural products from true mangrove flora: Source, chemistry and Bioactivities. Nat. Prod. Rep..

[36] Schwarz S., Noble W.C. (1999). Aspects of bacterial resistance to antimicrobials used in veterinary dermatological practice. Vet. Dermatol..

[37] Raut S.V., Anthappan P.D. (2013). Studies on antimicrobial activity of leaves extract of *Sonneratia alba*. Curr. Res. Microbiol. Biotechnol..

[38] Khafagi I., Gab-Alla A., Salama W., Fouda M. (2003). Biological activities and phytochemical constituents of the gray mangrove *Avicennia marina* (Forssk.) Vierh. Egypt. J. Biol..

[39] Mukhopadhyay A., Peterson R.T. (2006). Fishing for new antimicrobials. Curr. Opin. Chem. Biol..

[40] Hugo W.B., Russell A.D., Russell A.D., Hugo W.B., Ayliffe G.A.J. (1982). Types of antimicrobial agents. Principles and Practice of Disinfection, Preservation and Sterilization.

[41] Neu H.C. (1992). The crisis in antibiotic resistance. Science.

[42] Tenover F.C. (2006). Mechanisms of antimicrobial resistance in bacteria. Am. J. Med..

[43] Sikkema J., De Bont J.A., Poolman B. (1995). Mechanisms of membrane toxicity of hydrocarbons. Microbiol. Rev..

[44] Bajpai V.K., Sharma A., Baek K.H. (2015). Antibacterial mode of action of *Ginkgo biloba* leaf essential oil: Effect on morphology and membrane permeability. Bangladesh J. Pharmacol..

[45] Yenna T.W., Khanb M.A., Syuhadaa N.A., Ringa L.C., Ibrahimc D., Tan W.-N. (2017). Stigmasterol: An adjuvant for beta lactam antibiotics against beta-lactamase positive clinical isolates. Steroids.

[46] Wu H.-S., Raza W., Fan J.-Q., Sun Y.-G., Bao W., Shen Q.-R. (2008). Cinnamic acid inhibits growth but stimulates production of pathogenesis factors by in vitro cultures of *Fusarium oxysporum* f.sp. niveum. J. Agric. Food Chem..

[47] Korošec B., Sova M., Turk S., Kraševec N., Novak M., Lah L., Stojan J., Podobnik B., Berne S., Zupanec N. (2014). Antifungal activity of cinnamic acid derivatives involves inhibition of benzoate 4-hydroxylase (CYP53). J. Appl. Microbiol..

[48] Bogdanov S. (1997). Antibacterial substances in honey. Swiss Bee Res. Center.

[49] Mandal M.D., Mandal S. (2011). Honey: Its medicinal property and antibacterial activity. Asian Pac. J. Trop. Biomed..

[50] Johannes E., Litaay M., Syahribulan (2016). The bioactivity of hexadecanoic acid compound isolated from hydroid aglaophenia cupressina lamoureoux as antibacterial agent against *Salmonella typhi*. Int. J. Biol. Med. Res..

[51] Radiati L.E. (2002). Mechanism of virulence inhibition of enterophatogenic bacteria by ginger (*Zingiber officinale* Roscoe) rhizome extract. Ph.D. Dissertation.

[52] Grace O.O. (1989). Evaluation of the antimicrobial activity of citral. Lett. App. Microbiol..

