# Supplementary material for: Antibacterial and Antifungal Activity of the Extracts of Different Parts of Avicennia marina (Forssk.) Vierh
Source: Plants (Basel). 2021 Jan 28;10(2):252. doi: 10.3390/plants10020252 (PMC7911470; doi:10.3390/plants10020252)
Supplement: Supplementary file 1 [file plants-10-00252-s001.pdf]

Table S1. GC-MS based analysis of chemical composition of various plant parts of *Avicennia marina* plant by the various solvents

`

Sample	Molecule name	Retention time	formula
Leaves **(Ethyl acetate)**	1-TETRADECENE	13	C14H28
	2,4-BIS(1-METHYL-1-PHENYLETHYL)PHENOL OR 2,4-BIS(ALPHA,ALPHA-DIMETHYLBENZYL)PHENOL	32.5	[C6H5C(CH3)2]2C6H3OH
	DODECANE, 1,1'-OXYBIS- OR 1-DODECOXYDODECANE	35	C24H50O
	2,,6,10,14,18,22-TETRACOSAHEXAE	37.3	C30H50
	NORUNS-12-ENE	44.5	C29H48
**Fruits (Ethyl acetate)**	CYCLOHEXANONE	4.42	(CH2)5CO
	1-HEPTADECANOL	22.2	CH3(CH2)16OH
	3-EICOSENE, (E)- OR (E)-3-ICOSENE	26.5	C20H40
	TETRACOSANE	40.93	H(CH2)24H
	TETRATETRACONTANE	43.94	CH3(CH2)42CH3
	2,,6,10,14,18,22-TETRACOSAHEXAE	45.47	C30H50
	NONACOSANE	46.69	C29H60

	**TETRACONTANE**	**49.26**	**CH3(CH2)38CH3**
Roots **(Ethanol)**	GLYCERIN	7.78	C3H8O3
	CIS-CINNAMIC ACID	18.35	C9H8O2
	HEXADECANOIC ACID<br>OR<br>PALMITIC ACID	30.79	C16H32O2
	2,,6,10,14,18,22-TETRACOSAHEXAE	45.47	C30H50
	25-ETHYL-27-NORCHOLESTA-5,24(Z	51.76	
	2-FURANCARBOXALDEHYDE, 5-(HYDRXYMETHYL)-<br>OR<br>HYDROXYMETHYLFURFURAL (HMF)	12.97	
	1-DEOXY-D-ALTRITOL<br>OR<br>FUCITOL	23.19	
	1,2-BENZENEDICARBOXYLIC ACID<br>OR<br>PHTHALIC ACID	18.58	C8H6O4
	HEXADECANOIC ACID<br>OR<br>PALMITIC ACID	30.79	
	3,4,7,11,15-PENTAMETHYL	45.47	
	STIGMAST-5-EN-3-OL, (3.BETA.,24S)-<br>OR<br>BETA-SITOSTEROL	51.76	
	TARAXASTEROL	52.81	
Leaves **(Ethanol)**	1,2-BENZENEDICARBOXYLIC ACID<br>OR<br>PHTHALIC ACID	18.58	C8H6O4
	2,6-BIS(1,1-DIMETHYLETHYL)PHENOL<br>OR<br>2,6-DI-TERT-BUTYLPHENOL	23.99	C14H22O
	2-HEXADECEN-1-OL, 3,7,11,15,-TETRAMETHYL<br>OR<br>PHYTOL	25.29	C20H40O
	2,4-BIS(DIMETHYLBENZYL)-6-T-BUTYLPHENOL	32.66	C28H34O
	2,6,10-TRIMETHYL-2,6,10-DODECATRIENE	37.42	
	TRIMETHYL[4-(1,1,3,3,-TETRAMETHYLBUTYL) PHENOXY	44.81	

roots (chloroform)	**PENTANE, 2,3,3-TRIMETHYL**	**20.8**	
	1,2-BENZENEDICARBOXYLIC ACID<br>OR<br>PHTHALIC ACID	30.56	C8H6O4
	PENTADECANE	37.62	
	TETRACOSANE	40.93	H(CH2)24H
	EICOSENE, 9-OCTYL	43.94	
Leaves (chloroform)	1,2-BENZENEDICARBOXYLIC ACID<br>OR<br>PHTHALIC ACID	28.18	C8H6O4
	DODECANE, 1,1'-OXYBIS-<br>OR<br>1-DODECOXYDODECANE	30.56	C24H50O
	(2S,3R)-3-DIMETHYL-T-BUTYLSILO	43.94	---------
	TRANS-STIGMASTA-5,22-DIEN-3.BETA<br>OR<br>STIGMASTERIN<br>OR<br>STIGMASTEROL	46.69	C29H48O
	GAMMA-SITOSTEROL<br>OR<br>CLIONASTEROL	51.2<br>51.76	C29H50O
Fruits (Petroleum ether)	HEXADECANOIC ACID<br>OR<br>PALMITIC ACID	14.46	C16H32O2
	OCTADECANE	25.99	
	2,6,10,15,19,23-HEXAMETHYL-2,6	27.52	
	TETRATETRACONTANE	31.29.	CH3(CH2)42CH3
	TETRACONTANE	33.67	CH3(CH2)38CH3
